# Human articular chondrocytes produce IL-7 and respond to IL-7 with increased production of matrix metalloproteinase-13

**DOI:** 10.1186/ar2376

**Published:** 2008-02-20

**Authors:** David Long, Simon Blake, Xiao-Yu Song, Michael Lark, Richard F Loeser

**Affiliations:** 1 Section of Molecular Medicine, Department of Internal Medicine, Wake Forest University School of Medicine, Medical Center Blvd, Winston-Salem, North Carolina 27157, USA; 2 Centocor Inc., Great Valley Parkway, Malvern, Pennsylvania 19355, USA

## Abstract

**Introduction:**

Fibronectin fragments have been found in the articular cartilage and synovial fluid of patients with osteoarthritis and rheumatoid arthritis. These matrix fragments can stimulate production of multiple mediators of matrix destruction, including various cytokines and metalloproteinases. The purpose of this study was to discover novel mediators of cartilage destruction using fibronectin fragments as a stimulus.

**Methods:**

Human articular cartilage was obtained from tissue donors and from osteoarthritic cartilage removed at the time of knee replacement surgery. Enzymatically isolated chondrocytes in serum-free cultures were stimulated overnight with the 110 kDa α5β1 integrin-binding fibronectin fragment or with IL-1, IL-6, or IL-7. Cytokines and matrix metalloproteinases released into the media were detected using antibody arrays and quantified by ELISA. IL-7 receptor expression was evaluated by flow cytometry, immunocytochemical staining, and PCR.

**Results:**

IL-7 was found to be produced by chondrocytes treated with fibronectin fragments. Compared with cells isolated from normal young adult human articular cartilage, increased IL-7 production was noted in cells isolated from older adult tissue donors and from osteoarthritic cartilage. Chondrocyte IL-7 production was also stimulated by combined treatment with the catabolic cytokines IL-1 and IL-6. Chondrocytes were found to express IL-7 receptors and to respond to IL-7 stimulation with increased production of matrix metalloproteinase-13 and with proteoglycan release from cartilage explants.

**Conclusion:**

These novel findings indicate that IL-7 may contribute to cartilage destruction in joint diseases, including osteoarthritis.

## Introduction

The loss of cartilage matrix that occurs in osteoarthritis (OA) is associated with a disturbance in the balance of anabolic (synthetic) and catabolic (destructive) activities of the articular chondrocytes [1]. There is increasing evidence that cytokines, including IL-1, IL-6, and tumor necrosis factor (TNF)-α, play a role in matrix destruction by enhancing chondrocyte catabolic activity [2]. In addition to inducing matrix degrading enzymes directly, these cytokines can also act by stimulating production of additional proinflammatory cytokines. IL-6 is among the cytokines produced by chondrocytes after IL-1 stimulation [3-5]. These two cytokines have been shown to act synergistically to induce cartilage breakdown [6], suggesting that chondrocytes have the ability to respond to co-stimulation with multiple cytokine signals. A role for local production of cytokines in the joint destruction that occurs in rheumatoid arthritis (RA) is well established, and there is increasing evidence for the role of cytokines in OA [7]. Determining which cytokines are responsible for joint tissue destruction in arthritis is the subject of continuing research.

IL-7 is a cytokine that produces a diverse array of biologic effects. It was first described as a factor that promotes the growth of B cells in mice [8]. Since then, much of the work on IL-7 has been focused on its importance within the context of lymphocyte cell biology (for review [9,10]). IL-7 is required for survival of peripheral T lymphocytes, possibly through negative regulation of apoptosis in these cells. Other sites of IL-7 production include intestinal epithelial cells, keratinocytes, endothelial cells, smooth muscle cells, and fibroblasts [9].

IL-7 has also been studied within the context of RA [10]. It has been shown that IL-7 is produced at higher levels by fibroblast-like synoviocytes isolated from patients with RA and that stimulation of these cells with the proinflammatory stimuli IL-1 and TNF-α upregulated production of IL-7 [11]. Other cells of the synovial tissue, including synovial macrophages and synovial T cells, have been shown to respond to IL-7 stimulation with production of the inflammatory cytokines TNF-α and interferon-γ [12]. It has also been demonstrated that levels of IL-7 in synovial fluid are increased in patients with RA [13]. In addition, IL-7 has been shown to induce bone loss by promoting secretion of RANKL (receptor activator of nuclear factor-κB ligand), a cytokine responsible for the formation of osteoclasts, from T cells [14]. Collectively, these data point strongly to a role for IL-7 in inflammatory joint disease, but a potential role for IL-7 as a mediator of cartilage destruction has not been reported.

Fibronectin fragments have been detected in cartilage and synovial fluid samples from patients with RA or OA [15] and are thought to play a role in cartilage destruction in arthritis by stimulating chondrocytes to produce matrix metalloproteinases (MMPs) as well as multiple cytokines and chemokines, including IL-1, IL-6, IL-8, monocyte chemotactic protein-1, and growth-related oncogene family members [5,16,17]. In the present study, we screened for additional cytokines produced by chondrocytes in response to fibronectin fragment stimulation and identified IL-7. Levels of production were compared using human articular chondrocytes isolated from nonarthritic cartilage from young and old adults and from patients with OA. The role of IL-1 and IL-6 in stimulating chondrocyte IL-7 production was also determined, as was the ability of IL-7 to stimulate chondrocytes directly. The results suggest a potential role for IL-7 as a factor contributing to cartilage inflammation and destruction in arthritis.

## Materials and methods

### Materials

Recombinant human proteins (IL-6, soluble IL-6 receptor, IL-1β, and IL-7) were purchased from R&D Systems (Minneapolis, MN, USA). Human MMP-13 ELISA, Human IL-7 Quantikine High Sensitivity ELISA Kit, and Human IL-7 Biotinylated Fluorokine Kit were also from R&D Systems. Phospho-PYK-2 antibody was from BioSource (Camarillo, CA, USA). Total PYK2 antibody and 110 kDa fibronectin fragment were from Upstate Biotechnology (Lake Placid, NY, USA). IL-7 receptor primers and SybrGreen PCR Mastermix were from SuperArray Biosciences (Frederick, MD, USA). RayBio Human Inflammation Antibody Array III and Matrix Metalloproteinase Antibody Array were from Raybiotech (Norcross, GA, USA). IL-6 neutralizing antibody was produced by Centocor (Horsham, PA, USA). IL-1 receptor antagonist (Anakinra) was a gift from Amgen (Thousand Oaks, CA, USA). Nitrate/Nitrite Colorimetric Assay Kit was from Cayman Chemical (Ann Arbor, MI, USA).

### Tissue acquisition and chondrocyte cell culture

Human ankle and knee articular cartilage were obtained from tissue donors within 48 hours of death through the Gift of Hope Organ and Tissue Donor Network (Elmhurst, IL, USA) or from the National Disease Research Interchange (Philadelphia, PA, USA), in accordance with institutional protocol. Each donor specimen was graded for degenerative changes based on the 5-point Collins scale (0 to 4), as modified by Muehleman and coworkers [18]. The OA cartilage was discarded tissue obtained after knee replacement surgery. Cartilage was dissected from the joints and digested in a sequential manner with Pronase (Calbiochem, Gibbstown, NJ, USA) and then overnight with collagenase, as previously described [19]. Viability of isolated cells was determined using trypan blue, and cells were counted using a hemocytometer. Monolayer cultures were established by plating cells in six-well plates at 2 × 10^6 ^cells/ml in Dulbecco's modified Eagle's medium (DMEM)/Ham's F-12 medium supplemented with 10% fetal bovine serum. Plates were maintained for about 5 to 7 days, with feedings every 2 days until they reached 100% confluence prior to experimental use.

### Cartilage explant culture and stimulation

For explant cultures, full-thickness cartilage discs were obtained using a 4 mm biopsy punch. Explants were cultured for 72 hours in DMEM/Ham's F-12 (1/1) media supplemented with 1% mini-ITS+ (5 nM insulin, 2 μg/ml transferrin, 2 ng/ml selenous acid, 25 μg/ml ascorbic acid, and bovine serum albumin/linoleic acid at 420/2.1 μg/ml) for recovery. Wet weight of tissue was then measured and explants were cultured at one explant per well in a 12-well plate in 500 μl serum-free media for 72 hours of stimulation. Cartilage matrix proteoglycan degradation was estimated by measuring glycosaminoglycan (GAG) release into the media using the dimethylmethylene blue assay as previously described [19]. Nitric oxide release was estimated by measuring nitrate levels in the medium using a commercially available kit (Cayman Chemical). To test that the assay was working properly, we stimulated one set of explants with 10 ng/ml of IL-1β and detected 2.2 μmol/l nitrate per milligram wet weight of tissue.

### Chondrocyte stimulation

Medium was changed to serum-free DMEM/Ham's F-12 medium with antibiotics 18 hours (overnight) and again 2 hours before each experiment. Appropriate stimuli were then added to cells. The following standard concentrations were used for stimulation (unless otherwise indicated): 500 nmol/l fibronectin fragment, 10 ng/ml IL-1β, 10 ng/ml IL-6 plus 20 ng/ml soluble IL-6 receptor, and 10 ng/ml IL-7. Inhibitor concentrations were 100 μg/ml IL-1 receptor antagonist and 500 ng/ml IL-6 neutralizing antibody and, when used, these were added 1 hour before stimulation. In experiments measuring basal IL-7 production, medium was collected after 48 hours of incubation in serum-free conditions. When storage was necessary, 0.1% sodium azide was added to the medium before storage at 4°C.

### Antibody array

One milliliter of media was analyzed with the Human Inflammation Antibody Array III (Raybiotech), which can detect 40 different cytokines, or the Human Matrix Metalloproteinase Antibody Array (Raybiotech), which can detect seven MMPs and three tissue inhibitors of metalloproteinases (TIMPs). Both membranes were spotted in duplicate with cytokine or MMP-specific antibodies. Membranes were incubated with culture media and analyzed in accordance with the manufacturer's instructions.

### ELISA

Medium was analyzed with either the Human MMP-13 or Human IL-7 High Sensitivity ELISA (R&D Systems), in accordance with the manufacturer's instructions. The minimum detectable dose of IL-7 using this assay is reported as <0.1 pg/ml, with intra-assay and inter-assay precisions (coefficients of variation) of 8.0 to 9.4 and 7.3 to 10.3 when using cell culture supernates. For the MMP-13 ELISA, medium was routinely diluted to obtain values that would fall within the range of the standard curve.

### Immunoblotting

Cells were washed with phosphate-buffered saline and lysed with lysis buffer that contained 20 mmol/l Tris (pH 7.5), 150 mmol/l NaCl, 1 mmol/l EDTA, 1 mmol/l EGTA, 1% Triton X-100, 2.5 mmol/l tetrapyrophosphate, 1 mmol/l glycerol phosphate, 1 mmol/l Na_3_VO_4_, 1 μl/ml leupeptin, and 1 mmol/l phenylmethylsulfonyl fluoride. Lysates were centrifuged to remove insoluble material, and the soluble protein concentration was determined using BCA reagent (Pierce, Rockford, IL, USA). Samples containing equal amounts of total protein were separated by SDS-PAGE, transferred to nitrocellulose, and probed with anti-phospho-PYK2 antibody. Blots were then stripped and probed with anti-total-PYK2 antibody to confirm equal loading. Densitometry measurements were taken using Kodak 1D image analysis software.

### Real-time PCR analysis

Total RNA was isolated using the RNeasy Mini Kit (Qiagen, Valencia, CA, USA). RNA from 10 different chondrocyte cultures was pooled and genomic DNA contamination was removed using Turbo DNA-free kit (Ambion, Austin, TX, USA), in accordance with the manufacturer's instructions. Two micrograms of DNA-free, pooled RNA was reverse transcribed using an AMV reverse transcriptase and oligo dT primer at 42°C for 1 hour. Two microliters of RT reaction was then combined in a reaction mixture with 1 μl specific primer pair, 12.5 μl 2× SybrGreen PCR Mastermix, and water to a final reaction volume of 25 μl. Reactions were then run in triplicate with 40 cycles of amplification on an ABI Prism 7000 real-time PCR machine (Applied Biosystems, Foster City, CA, USA). A negative control was included that contained primers, water and Mastermix but no cDNA, and another negative control was included that contained RNA that had not been reverse transcribed in order to detect contaminating genomic DNA. An amplification plot was generated using the ABI software. PCR specificity was confirmed by dissociation curve analysis (data not shown).

### IL-7 binding assay

For flow cytometry analysis, chondrocytes were removed from six-well dishes by trypsin digestion and for confocal microscopy analysis chondrocytes were examined directly in six-well dishes. In both instances, cells were stained with fluorescently labeled IL-7 using the Human IL-7 Biotinylated Fluorokine Kit (R&D Systems), in accordance with the manufacturer's instructions but with slight modifications. Briefly, cells were washed twice with phosphate-buffered saline, followed by incubation for 1 hour at 4°C with either 60 μl of biotinylated IL-7 or 60 μl of biotinylated negative control reagent or 60 μl biotinylated IL-7 complexed with a blocking antibody diluted in wash buffer. Avidin-fluorescein 60 μl was then added to each set of cells and incubation was continued for a further 30 minutes at 4°C. Cells were then washed three times with wash buffer and examined by either flow cytometry or confocal microscopy for green fluorescence using lasers with 488 nm excitation and 530 nm emission wavelengths.

### Statistical analysis

Unless indicated otherwise, results were analyzed using the Student's *t*-test in StatView 5.0 (SAS Institute Inc., Cary, NC, USA).

## Results

### Chondrocytes produce IL-7 in response to fibronectin fragment stimulation, aging, and OA

**Figure 1:**
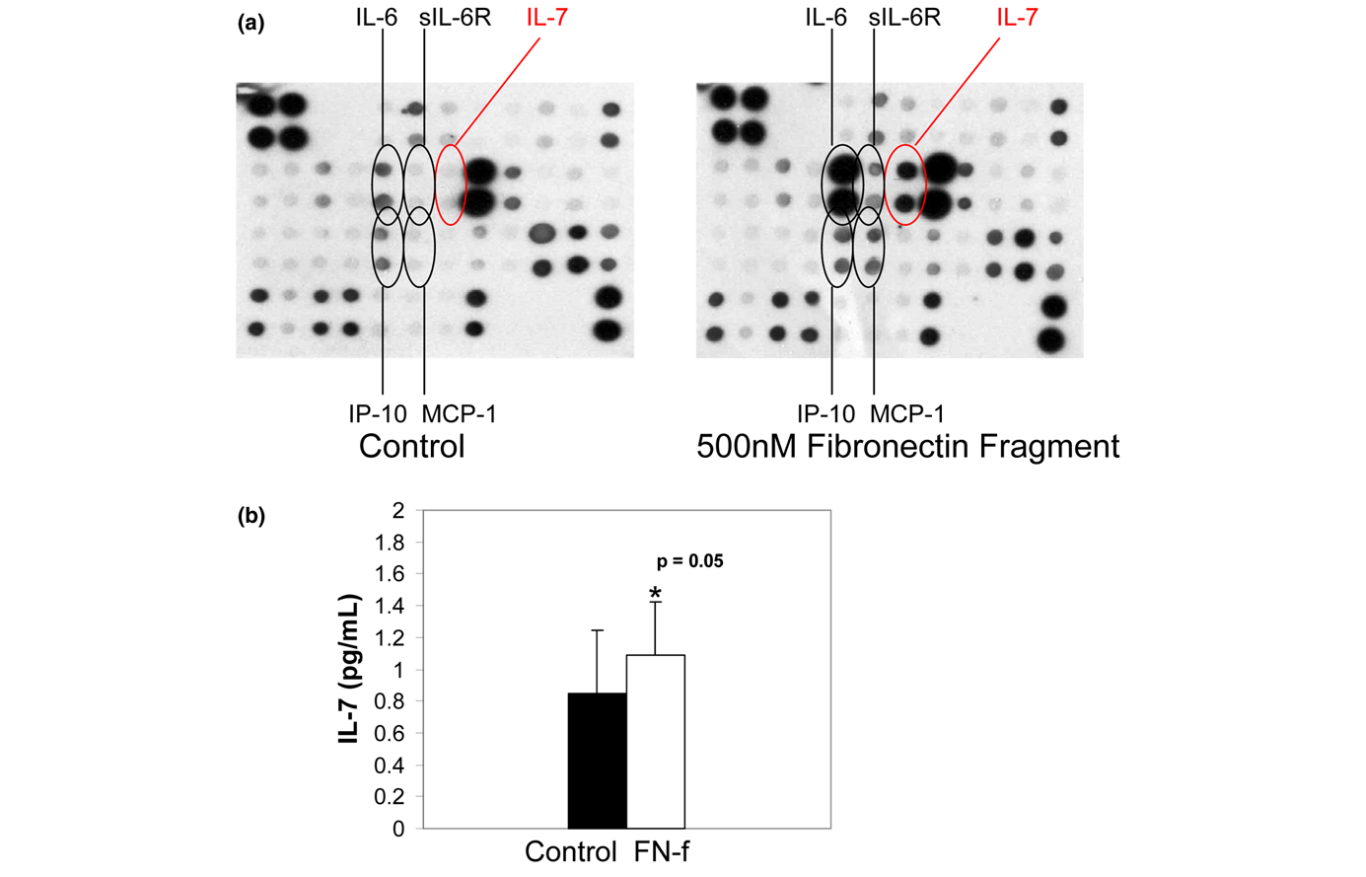
Chondrocytes produce IL-7 in response to stimulation with fibronectin fragments. Human articular chondrocytes obtained from normal articular cartilage and cultured in serum-free media were treated overnight with 500 nmol/l of the 110 kDa fibronectin fragment (FN-f). Media was collected and analyzed for cytokine production using **(a) **an inflammation antibody array or **(b) **an IL-7 ELISA. Results are representative of three experiments for each result with different donor cells used in each experiment. The IL-7 spots on the array are shown in the red circles. (Other spots that were shown to change after fibronectin fragment stimulation included IL-6, soluble IL-6 receptor [sIL-6R], interferon-inducible protein [IP]-10, and monocyte chemotactic protein [MCP]-1.)

**Figure 2:**
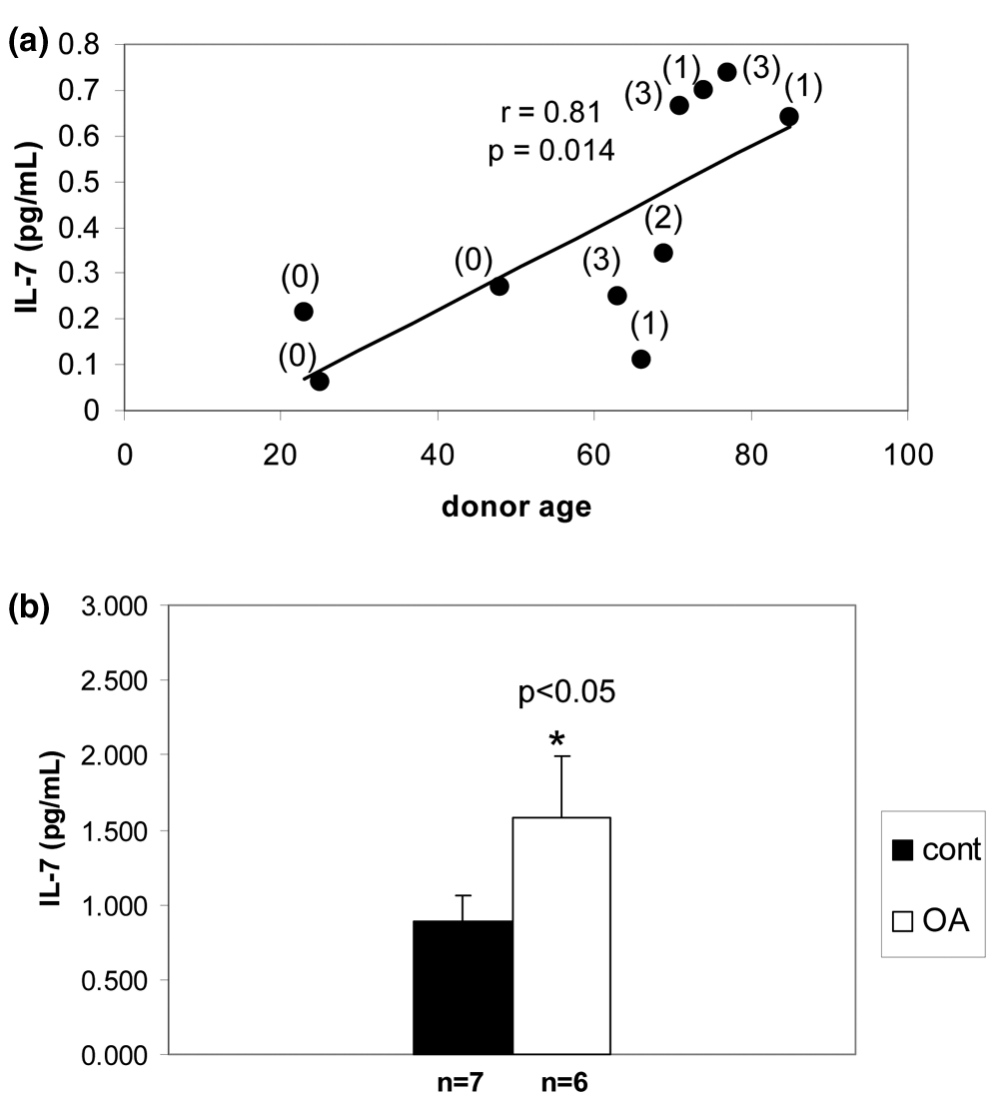
Effects of age and OA on chondrocyte production of IL-7. Media was collected 48 hours after changing to serum-free conditions in chondrocyte cultures established from **(a) **nonarthritic cartilage from 10 donors of different ages or from **(b) **cartilage from age-matched nonarthritic (*n *= 7) and osteoarthritic cartilage (*n *= 5). IL-7 was measured in the media using ELISA. The relationship of age to IL-7 levels was analyzed by Spearman correlation. The numbers in parentheses above the data points in panel a are the Collin's scores for the donor samples. OA, osteoarthritis.

Using an antibody array method, one of the cytokines found to be increased by fibronectin fragment stimulation was IL-7 (Figure 1a). This finding was confirmed by ELISA using additional chondrocyte cultures (Figure 1b). In previously published work, we showed that IL-1 production by chondrocytes increases with increasing donor age [20]. Using the IL-7 ELISA, we also found a significant (r = 0.818, *P *= 0.014) increase with age in the endogenous production of IL-7 by chondrocytes cultured for 48 hours in serum-free medium (Figure 2a). Although the younger donors all had Collin's scores of 0, a correlation between Collin's score and IL-7 levels was not evident in the older donors.

We also considered the possibility that IL-7 production by chondrocytes might be increased in cells isolated from OA cartilage. A significant (*P *< 0.05) increase in the production of endogenous IL-7 by isolated OA chondrocytes cultured in serum-free medium was noted when compared with cells from age-matched nonarthritic cartilage (Figure 2b).

### Chondrocytes express the IL-7 receptor

**Figure 3:**
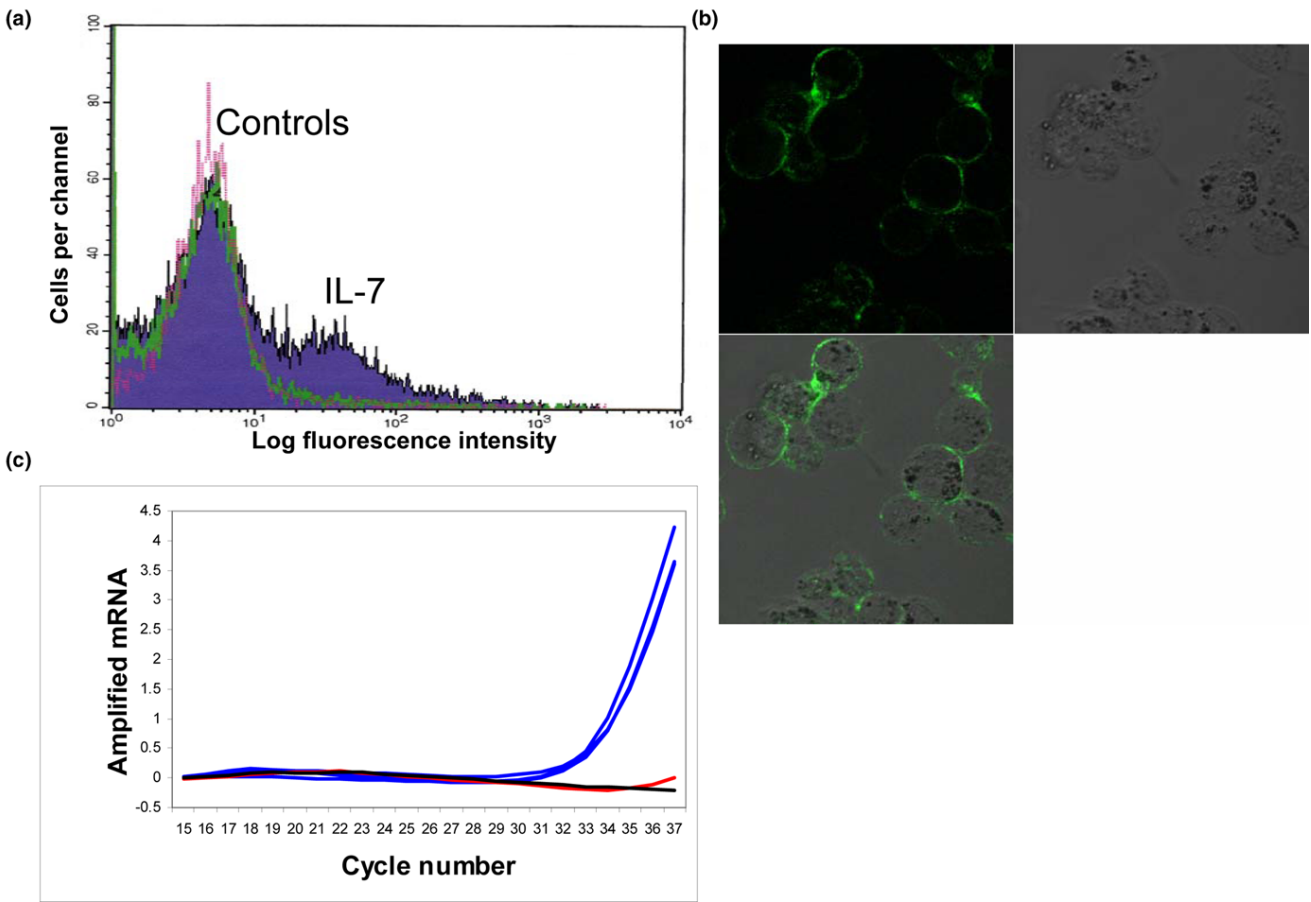
Chondrocyte expression of IL-7 receptors. **(a) **Chondrocytes isolated from normal cartilage (*n *= 1) were incubated with a fluorescently labeled recombinant IL-7 to demonstrate binding of IL-7 to the cell surface. Labeled cells were examined by flow cytometry. The peak that is shaded purple with the black line shows cells stained with IL-7, the peak with the pink line shows blocking antibody negative control, and the peak with the green line shows cells stained with the biotin negative control. **(b) **Chondrocytes isolated from normal cartilage were incubated with a fluorescently labeled recombinant IL-7 as above. Labeled cells were examined by confocal microscopy. IL-7 staining is shown in green. Top left is the green channel, top right is differential intermittent contrast, and bottom left is the merged image. Chondrocytes from eight different donors showed similar results. **(c) **Pooled RNA isolated from 10 different sets of cultured chondrocytes was subjected to reverse transcription and real-time PCR with an IL-7 receptor primer set. An amplification plot is shown to demonstrate positive signal. Amplified chondrocyte cDNA in triplicate is shown with the blue lines. Negative control with no reverse transcription of RNA before real-time PCR is shown with a red line. Negative control with no cDNA is shown with the black line.

Having shown that chondrocytes can produce IL-7, we next wished to determine whether IL-7 could be acting in an autocrine or paracrine fashion in cartilage. Using fluorescently labeled IL-7, examination by either flow cytometry (Figure 3a) or confocal microscopy (Figure 3b) detected fluorescent IL-7 bound to chondrocytes. Similar results were noted using a monoclonal antibody to the IL-7 receptor (data not shown). IL-7 receptor expression by chondrocytes was also confirmed by real-time PCR using RNA isolated from cartilage of 10 different tissue donors (Figure 3c). Taken together, these lines of evidence suggest that chondrocytes express the IL-7 receptor and thus might be capable of responding to IL-7 in an autocrine or paracrine fashion.

### Chondrocytes respond to IL-7 stimulation

**Figure 4:**
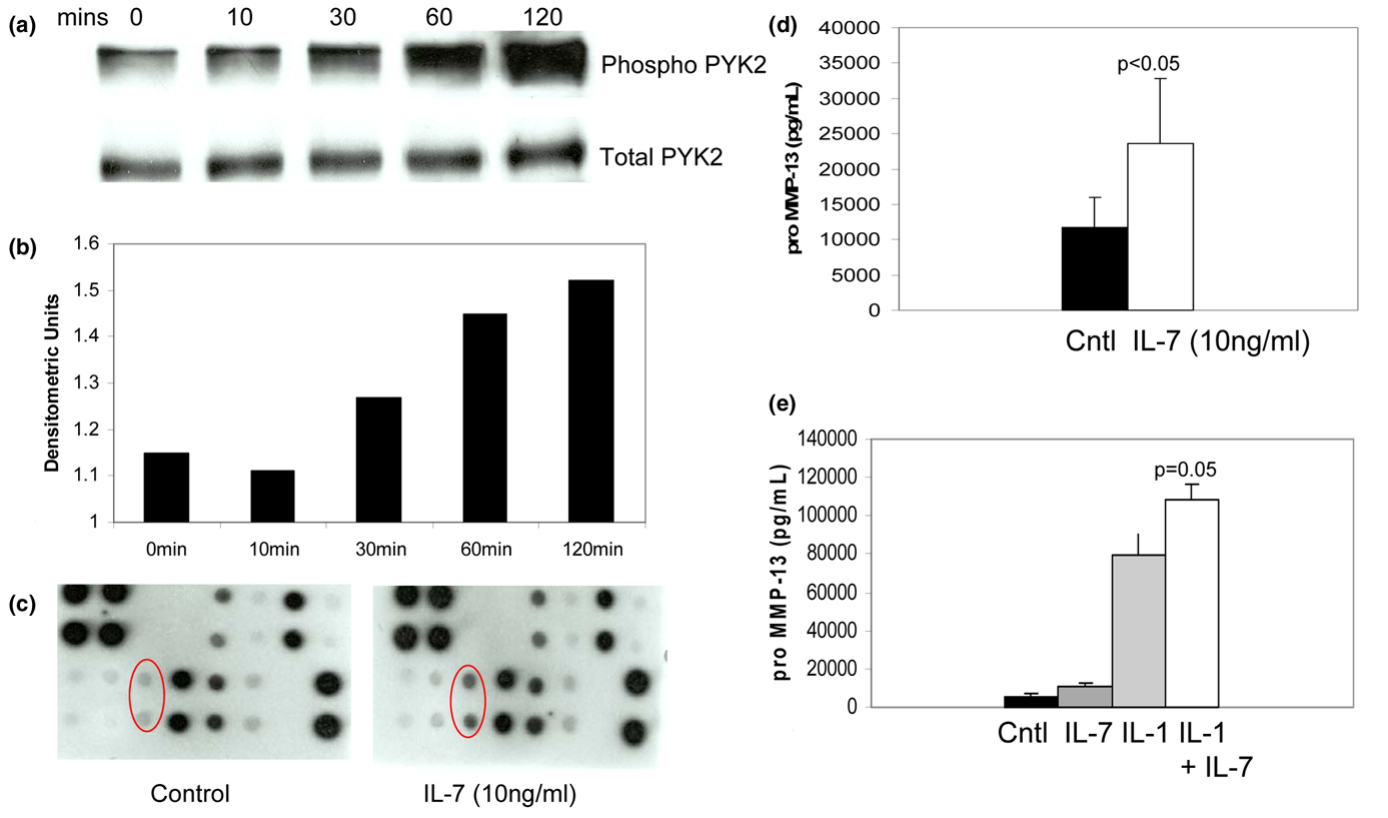
Chondrocytes respond to IL-7 stimulation with increased PYK2 phosphorylation and production of MMP-13. **(a) **Chondrocytes isolated from normal adult cartilage were stimulated with 10 ng/mL recombinant IL-7 and lysates were made at indicated time points for immunoblotting with an antibody to phosphorylated proline-rich tyrosine kinase (PYK)2 (Tyr402). The blot was then stripped and probed with total PYK2 antibody to confirm equal loading. **(b) **Densitometric scanning of the blot shown in panel a. **(c) **Medium was collected from serum-free chondrocyte cultures after overnight stimulation with 10 ng/ml recombinant IL-7 and examined for the presence of multiple matrix metalloproteinase (MMP) family members using an MMP antibody array. MMP-13 spots are shown in circles. **(d,e) **Media was collected from serum-free chondrocyte cultures after overnight stimulation with 10 ng/ml recombinant IL-7 or IL-1β, or the two together, and examined for the presence of MMP-13 using a commercially available ELISA. Results are the mean of seven experiments.

Proline-rich tyrosine kinase (PYK)2 is a nonreceptor tyrosine kinase that was previously shown to be activated in response to IL-7 stimulation [21], and we previously showed that activation of PYK2 is required for chondrocyte fibronectin fragment stimulated MMP-13 production [22]. Therefore, we wished to determine whether PYK2 would be phosphorylated by chondrocytes in response to IL-7 stimulation. In initial experiments, chondrocytes were stimulated with 100 ng/ml recombinant IL-7 and cells were lysed at different time points over the course of 2 hours. PYK2 phosphorylation was noted by 30 minutes and reached a maximum at 2 hours (Figure 4a). The experiment was repeated using a 10 ng/ml concentration of IL-7 with similar results (data not shown).

We next determined whether IL-7-mediated PYK2 phosphorylation was associated with production of matrix-degrading enzymes, as we had previously shown using fibronectin fragment stimulation. We chose a 10 ng/ml dose of IL-7 for further experiments, based on previous dose-response studies conducted in other cell types that found that 10 ng/ml was required for stimulation of mononuclear and T-cell proliferation [11,13] and TNF-α production [12]. Chondrocytes were treated overnight with recombinant IL-7, and MMP secretion into the media was analyzed with an MMP antibody array that included MMP-1, -2, -3, -8, -9, -10 and -13, as well as TIMP-1, -2 and -4. Interestingly, the only MMP on the array found to be increased after IL-7 stimulation was MMP-13 (Figure 4b), which suggests that IL-7 may be acting through a pathway different from those employed by other catabolic cytokines, which upregulate multiple MMPs. None of the TIMPs were increased after IL-7 stimulation. The IL-7 stimulation of MMP-13 production was confirmed by ELISA using additional chondrocyte cultures (Figure 4c). In cultures from three donors, we also tested IL-7 at 0.1 ng/ml and found an almost twofold increase in MMP-13 (data not shown). Although IL-7 has been shown to stimulate TNF-α production by monocytes and CD4^+ ^T cells [12], we could not detect, by ELISA, TNF-α in media from chondrocytes after overnight stimulation with IL-7 (data not shown).

Several cytokines have been shown to act synergistically with IL-1 to increase MMP-13 production. We therefore wished to examine the ability of IL-7 to act synergistically with IL-1. As shown in Figure 4c, IL-7 was not as potent as IL-1β but the combination of IL-1 and IL-7 increased MMP-13 levels in the media to a greater extent than did IL-1 treatment alone.

### IL-7 causes proteoglycan release from cartilage explants

**Figure 5:**
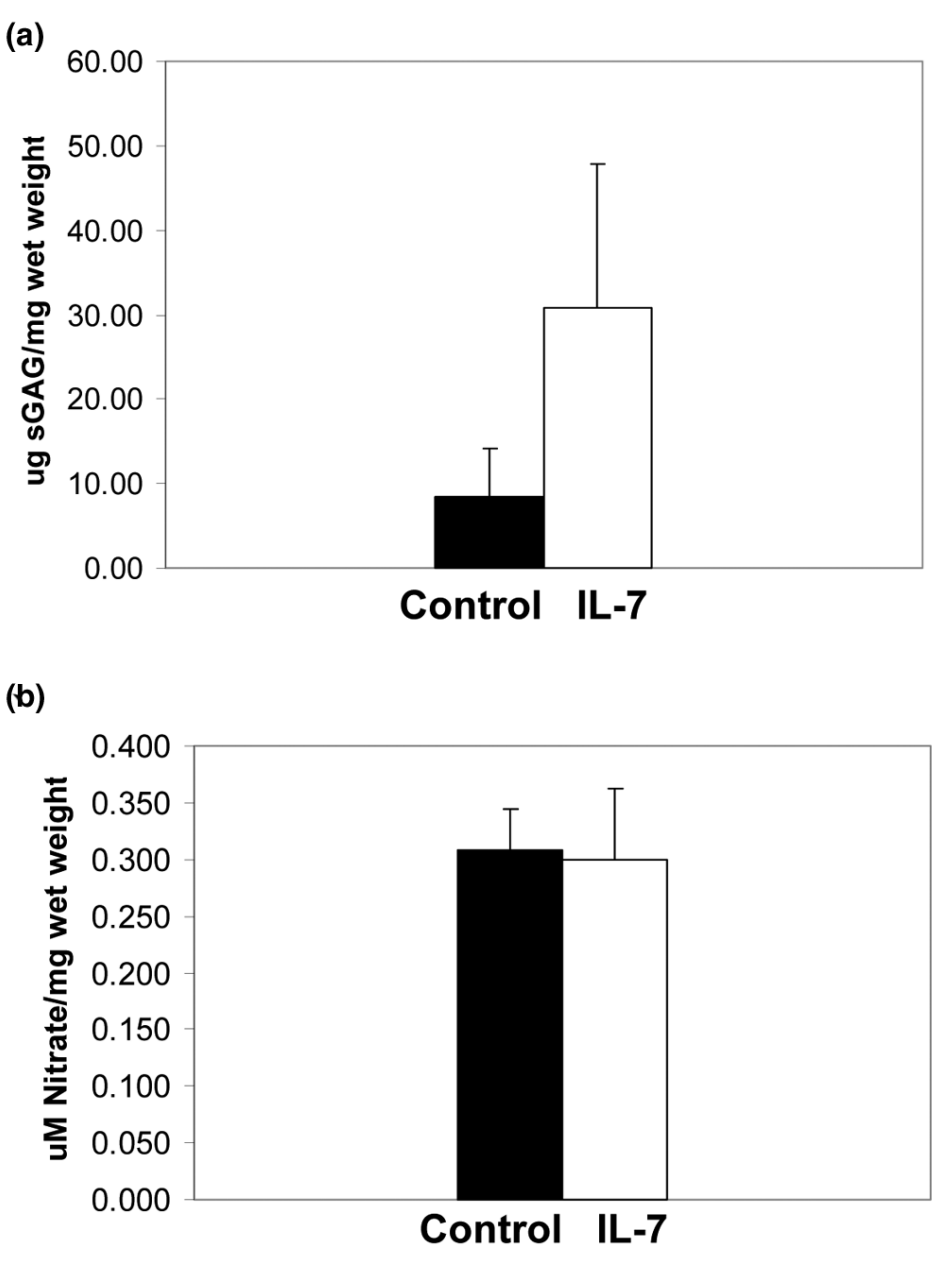
IL-7 causes proteoglycan release, but not nitric oxide production, in cartilage explants. Cartilage explants were stimulated for 72 hours with 10 ng/ml recombinant human IL-7 before media collection. **(a) **Medium was analyed for sulfated glycosaminoglycan (sGAG) using the dimethylmethylene blue assay and normalized for the wet weight of the tissue. **(b) **Total nitrite was measured in the media as a marker for nitric oxide production using commercially available colorimetric nitrate/nitrite assay kit. Results represent four experiments.

In order to further determine whether IL-7 might serve as a catabolic mediator in articular cartilage, we stimulated cartilage explants with 10 ng/ml IL-7 for 72 hours and measured GAG release in the medium. Indeed, IL-7 caused a significant increase in GAG release from cartilage explants relative to controls (Figure 5a). Increased production of nitric oxide by chondrocytes is also a characteristic of several catabolic cytokines, including IL-1, but – unlike in explants treated with IL-1β – we did not detect an increase in nitrate levels in media from explants treated with IL-7 (Figure 5b).

### The combination of IL-1 and IL-6 stimulates production of IL-7 by chondrocytes

**Figure 6:**
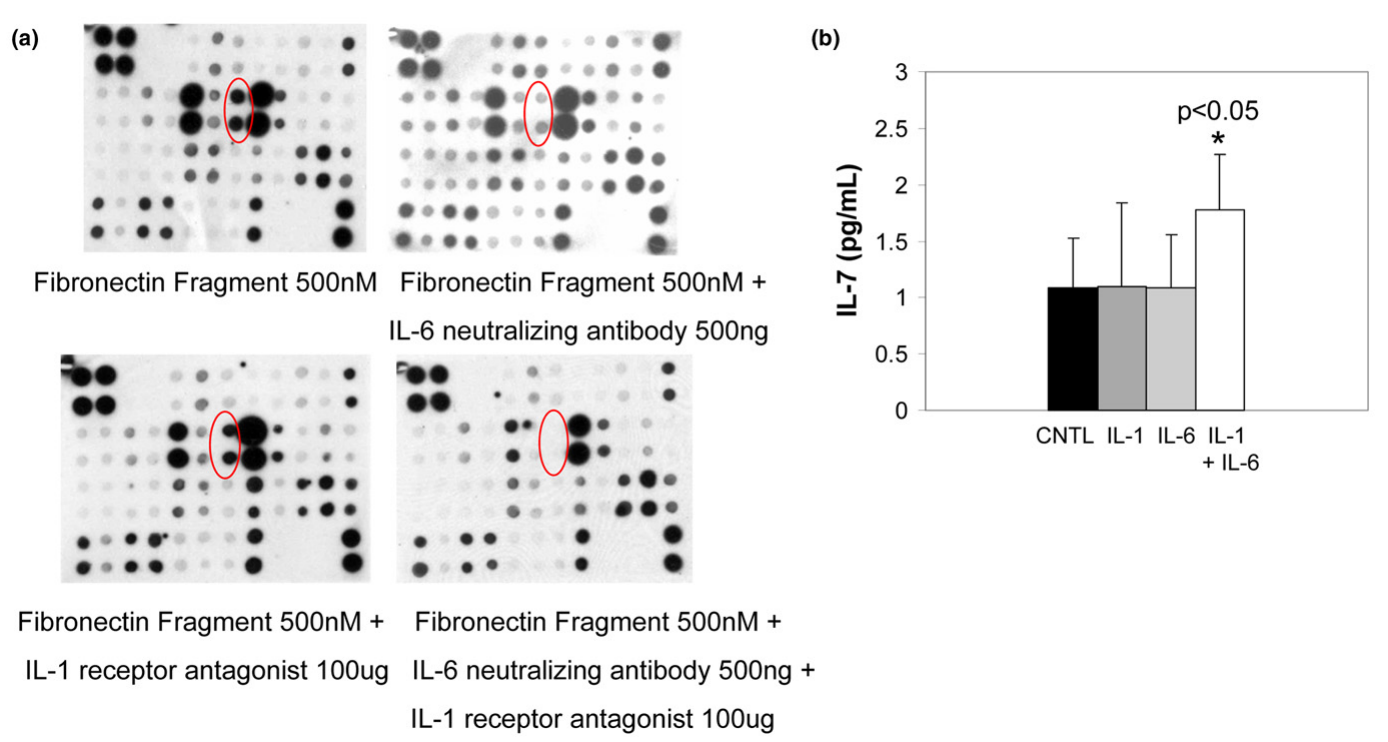
Role for IL-1 and IL-6 in stimulation of IL-7 production by chondrocytes. **(a) **Chondrocytes were pretreated with either an IL-6 neutralizing antibody or the IL-1 receptor antagonist, or the combination of the two inhibitors, and then subsequently stimulated with fibronectin fragment. After overnight stimulation media samples were collected and used for an inflammation antibody array. IL-7 spots are shown in red circles. **(b) **Chondrocytes were stimulated with either IL-1β (10 ng/ml) or IL-6/soluble IL-6 receptor (10 ng/ml and 20 ng/ml) or the combination of cytokines. Medium was collected and subsequently analyzed with an IL-7 ELISA.

In previous studies we demonstrated that chondrocyte fibronectin fragments stimulation increased production of several cytokines and chemokines, including IL-1β and IL-6 [5], which might be responsible for inducing IL-7 production in an autocrine/paracrine manner. Therefore, chondrocytes were pretreated for 1 hour with either 100 μg/ml IL-1 receptor antagonist or 500 ng/ml IL-6 neutralizing antibody, or the combination of both, before addition of fibronectin fragments. IL-6 neutralizing antibody alone reduced fibronectin fragment stimulated IL-7 production, whereas the IL-1 receptor antagonist showed no inhibition (Figure 6a). However, when both inhibitors were added together, the combination completely blocked IL-7 production (Figure 5a). This suggested that chondrocyte IL-7 production was a result of the combined effects of IL-1 and IL-6. To test this hypothesis, chondrocytes were stimulated overnight with either recombinant IL-1β, IL-6 plus soluble IL-6 receptor (necessary to stimulate chondrocytes with IL-6), or the combination of the cytokines. Indeed, the combination of the cytokines together was required to induce IL-7 production (Figure 6b). These results suggest a role for co-stimulation of chondrocyte IL-7 in response to IL-1 and IL-6.

## Discussion

Although IL-7 has traditionally been thought of as a T-cell regulatory cytokine, in this report the ability of human articular chondrocytes to produce IL-7, express an IL-7 receptor, and respond to IL-7 stimulation was demonstrated. Chondrocyte production of IL-7 was stimulated by catabolic and proinflammatory mediators, including the 110 kDa fibronectin fragment, and by the combined actions of IL-1β and IL-6. The stimulation of chondrocyte IL-7 production by fibronectin fragments appeared to be part of an autocrine loop mediated by the fragment stimulation of IL-1 and IL-6 production, because inhibition of these cytokines blocked fragment stimulated IL-7 production. IL-7 stimulated chondrocytes to produce MMP-13, a metalloproteinase that is responsible for degradation of type II collagen in cartilage, and caused proteoglycan release from cartilage explants. Additionally, increased production of IL-7 was measured in cultures of osteoarthritic chondrocytes relative to normal chondrocytes. These findings suggest a potential involvement of IL-7 in the OA disease process.

To our knowledge, this is the first report of IL-7 protein production and IL-7 receptor expression by articular chondrocytes. A previous study used RT-PCR to detect IL-7 RNA in human articular cartilage obtained from patients with RA but could not detect IL-7 message in OA or normal cartilage [23]. A second RT-PCR study confirmed IL-7 expression in RA cartilage but also detected IL-7 message in two out of six cartilage samples from OA patients, one out of five cartilage samples from infants, and in all seven cartilage samples from mice aged 4–8 days [24]. Mean levels of IL-7 in synovial fluid, measured using ELISA, were reported to be 34 pg/ml in 44 RA patients and 1.1 pg/ml in 10 patients with OA [13].

Based on the results from the inflammation antibody array (Figure 1a), we expected to find significantly higher levels of IL-7 than the low pg/ml range measured using the ELISA. The reason for this discrepancy is not clear but could be due to the different antibodies used to detect IL-7 in the two assays, or perhaps the presence of binding molecules, such as soluble IL-7 receptor or proteoglycans, that might have affected the ELISA measurement differently from the membrane array. However, the 1 to 2 pg/ml amount of IL-7 we detected in chondrocytes stimulated with fibronectin fragments or IL-1 plus IL-6 is higher than the 0.33 pg/ml IL-7 reported to be produced by cultured RA synovial fibroblasts and is the same as the amounts made by these cells after stimulation with IL-1β or TNF-α [11].

The highest levels of IL-7 were noted in cultured cells established from the cartilage of older tissue donors. In previous work [20] we also noted an age-related increase in production of IL-1β as well as increased production of MMP-13 in response to IL-1 or fibronectin fragments. These findings suggest an age-related increase in the proinflammatory environment of cartilage that could contribute to cartilage destruction and the development of arthritis in older adults.

In addition to the demonstration that chondrocytes express IL-7 receptors and produce MMP-13 when cultured in the presence of IL-7, the ability of chondrocytes to respond to IL-7 (10 ng/ml) was demonstrated by examining phosphorylation of a nonreceptor tyrosine kinase, namely PYK2. Activation of PYK2 through IL-7 stimulation (50 ng/ml) was previously reported in thymocytes [21]. Signaling mediated by PYK2 in chondrocytes appears to be an important component of several catabolic pathways. In addition to a role in fibronectin fragments mediated MMP-13 production [22], PYK2 has been shown to be involved in MMP-13 production by chondrocytes stimulated with the inflammatory protein S100A4 through a pathway involving intracellular calcium and reactive oxygen species [25]. It has also been shown to be involved in chondrocyte production of nitric oxide and MMP-3 induced by monosodium urate monohydrate crystals [26].

Many cytokines have been identified as secretion products of chondrocytes and their role in OA has become a subject of increasing interest [2,7]. Increased local cytokine activity may also play an important role in the cartilage destruction that occurs in RA. The principal cytokines receiving the most attention to date as mediators of cartilage destruction have been IL-1β and TNF-α. However, chondrocytes have been shown to produce a host of cytokines and inflammatory mediators, many of which are also produced by monocytes/macrophages [27]. IL-7 can be added to this list of mediators. IL-7 is unlikely to be a sole mediator of cartilage destruction in arthritis. However, because IL-7 can stimulate cells to produce additional cytokines, such as IL-6, IL-8 and TNF-α [10] and (as shown here) can stimulate additional production of MMP-13 when combined with IL-1β, it may be an important contributor to joint tissue destruction in OA and RA.

## Conclusion

IL-7 can be produced by articular chondrocytes, which also express IL-7 receptors. Production of IL-7 is increased in chondrocytes from older donors, from OA cartilage, and after stimulation with fibronectin fragments, IL-1, and IL-6. Treatment of chondrocytes with IL-7 stimulates PYK2 phosphorylation, increases the production of MMP-13, and results in GAG release from cartilage explants. These findings suggest that IL-7 may contribute to matrix destruction in arthritis.

## Abbreviations

DMEM = Dulbecco's modified Eagle's medium; ELISA = enzyme-linked immunosorbent assay; GAG = glycosaminoglycan; IL = interleukin; MMP = matrix metalloproteinase; OA = osteoarthritis; PCR = polymerase chain reaction; PYK = proline-rich tyrosine kinase; RA = rheumatoid arthritis; RT = reverse transcription; TIMP = tissue inhibitor of metalloproteinases; TNF = tumor necrosis factor.

## Competing interests

Richard Loeser received a research grant from Centocor. Simon Blake, Xiao-Yu, and Michael Lark are employees of Centocor and own stock in the company.

## Authors' contributions

DL designed and carried out experiments and helped to draft the manuscript. SB, X-YS, and ML contributed to the design of the study and interpretation of data. RL contributed to study design, supervised the performance of experiments, interpreted data, and completed the writing of the manuscript. All authors approved the content of the manuscript.
